# PAIN AND MEMORY LANE: SEX-SPECIFIC INSIGHTS INTO STRESS REACTIVITY, PAIN, AND COGNITION IN MID- TO LATE LIFE

**DOI:** 10.1093/geroni/igae098.0399

**Published:** 2024-12-31

**Authors:** Ashley Curtis, Amy Costa, Melanie Stearns, Kevin McGovney, Mary Beth Miller, Christina McCrae

**Affiliations:** University of South Florida, Tampa, Florida, United States; University of South Florida, Tampa, Florida, United States; University of South Florida, Tampa, Florida, United States; University of Missouri, Columbia, Missouri, United States; University of Missouri, Columbia, Missouri, United States; University of South Florida, Tampa, Florida, United States

## Abstract

Pain and cognition are bidirectionally associated. Perceived stress reactivity is linked to pain and cognitive perception, but interactive relationships are unclear. Sex differences are established in all factors (pain/stress reactivity/cognition), but studies in aging and chronic pain populations are limited. This study examined sex differences in interactive associations of pain and stress reactivity on memory in middle-aged/older adults with chronic pain. Cognitively healthy aging adults (N=678; 52%-women; Mage=62.4±11.3) experiencing frequent pain in past 3 months completed assessments of pain intensity (average past week), stress reactivity (Perceived Stress Reactivity Scale), and memory (Everyday Memory Questionnaire). Multiple regressions evaluated interactions between sex, pain, and stress reactivity on memory, controlling for age, education, #medical conditions, sleep/pain medication, and depression. Sex interacted with pain in associations between stress reactivity and memory (R-squared=.005, p=.04). Greater stress reactivity was associated with worse memory for women with lowest pain (~22/100; B=.59±.24, p=.01), both women (B=.63±.18, p<.001) and men (B=.76±.20, p<.001) with intermediate pain (~48/100), and both women (B=.67±.23, p=.003) and men (B=1.30±.28, p<.001) with highest pain (~73/100). Findings suggest relatively consistent associations between stress reactivity and memory across pain levels in aging women. In aging men, stress reactivity impacts memory at moderate pain levels, but this is exacerbated (two-fold) as pain increases. Sex-specific tailoring of behavioral pain treatments is encouraged. Techniques to reduce stress reactivity (cognitive control, mindfulness) may benefit memory. Prospective studies and objective assessments of stress reactivity, pain, cognition, and circulating sex hormones are needed to understand sex-specific modifiable risk factors of cognitive decline.

